# 2-Methoxy-7-Acetonyljuglone Isolated from *Reynoutria japonica* Increases the Activity of Nuclear Factor Erythroid 2-Related Factor-2 through Inhibition of Ubiquitin Degradation in HeLa Cells

**DOI:** 10.3390/antiox8090398

**Published:** 2019-09-14

**Authors:** Jung-Hwan Kim, Atif Ali Khan Khalil, Hye-Jin Kim, Sung-Eun Kim, Mi-Jeong Ahn

**Affiliations:** 1 Department of Pharmacology, School of Medicine, Institute of Health Sciences, Gyeongsang National University, Jinju 52727, Korea; 2 College of Pharmacy and Research Institute of Pharmaceutical Sciences, Gyeongsang National University, Jinju 52828, Korea; atif.khalil7799@gmail.com (A.A.K.K.); black200203@gmail.com (H.-J.K.); 3 Department of Food and Nutrition, Sookmyung Women’s University, Yongsan-gu, Seoul 04310, Korea; sekim@sookmyung.ac.kr

**Keywords:** NRF2, 2-methoxy-7-acetonyljuglone, antioxidant, ubiquitination

## Abstract

The nuclear factor erythroid-derived 2-related factor 2 (NRF2) is a key transcription factor for the activation of genes responsible for oxidative stress and drug detoxification. Thus, it is important to identify NRF2 activators, which can be used to protect the cells from oxidative damage. Here, we investigated the effect of juglone derivatives isolated from *Reynoutria japonica* on the activity of NRF2 in HeLa cells. We demonstrated that among the juglone derivatives, 2-methoxy-7-acetonyljuglone (MA) strongly stimulated the antioxidant response element (ARE)-luciferase activity in a dose-dependent manner. In addition, MA significantly increased the nuclear localization of NRF2 and, consequently, increased the expression of NRF2 target genes, including heme oxygenase-1(*HO-1*), NAD(P)H: quinine oxidoreductase-1 (*NQO-1)*, and glutamate-cysteine ligase catalytic (*GCLC*). To gain insights into the NRF2 signaling mechanism by MA, we measured the activities of RAC-alpha serine/threonine-protein kinase (AKT) and mitogen-activated protein (MAP) kinase family proteins, including extracellular signal-regulated kinase (ERK) and p38. Our results showed that MA induced NRF2 activity through p38 and AKT signaling. Subsequently, we found that MA significantly enhanced NRF2 stability by inhibiting ubiquitin-dependent proteasomal degradation. Thus, MA might protect cells by enhancing the activity and stability of NRF2 through inhibition of the proteasomal degradation pathway.

## 1. Introduction 

Nuclear factor erythroid 2-related factor 2 (NRF2) is a transcription factor that activates the expression of genes responsible for antioxidative stress and drug detoxification. The role of NRF2 has been implicated in stress-induced pathophysiological problems, including inflammatory responses, cardiovascular diseases, age-related diseases, and many cancers. The function of cytosolic NRF2 is regulated by Kelch-like erythroid cell-derived protein with CNC homology [ECH]-associated protein (Keap1), which is an inhibitory factor of NRF2 [1,2]. Under oxidative or electrophilic stress conditions, stabilized NRF2 translocates into the nucleus by dissociating itself from Keap1 and heterodimerizes with a small Maf protein in the nucleus. Subsequently, the transcriptional complex can bind to the antioxidant response element (ARE) found in the promoters of the target genes and regulate the expression of these genes [3].

The antioxidant response element (ARE) is a *cis*-acting enhancer DNA sequence found in the promoters of several genes encoding antioxidants and phase II detoxification enzymes. Several stimuli, including oxidants, electrophiles, and natural products can activate the ARE and expression of the respective genes [1,4,5,6]. NRF2 controls the expression of antioxidant enzymes such as glutamate-cysteine ligase catalytic (GCLC), heme-oxygenase-1 (HO-1), and NAD(P)H: quinine oxidoreductase-1 (NQO-1). Among these, HO-1 is a major antioxidant enzyme that plays an important role in protecting cells from oxidative stress and inflammation. Expression of these genes is induced by NRF2 through its interactions with the ARE enhancer sequence [7,8]. Additionally, many signaling molecules are known to increase the transcriptional activity of NRF2 during expression of its target genes [9,10].

Several factors modulate the function of NRF2, for instance, modification of Keap1 [1], phosphorylation of NRF2 by mitogen-activated protein kinases (MAPKs), cyclic AMP-activated protein kinase α (AMPKα) and phosphatidylinositol 3-kinase (PI3K)/AKT [9,10] as well as the NRF2 stability by GTPase-activating-like protein 1 (IQGAP1) [11].

It has been reported that natural substances can regulate the NRF2 activity through a variety of molecular mechanisms, including phosphorylation of serine/threonine residues by mitogen-activated protein kinases (MAPKs), cAMP-activated protein kinase (AMPK), AKT, and protein kinase C (PKC) [12,13,14,15]. In addition, natural compounds can stimulate NRF2 activation through the modification of Keap1 [12,13,14,15,16].

*Reynoutria japonica* Houtt., Japanese knotweed, is a medicinal plant and belongs to the Polygonaceae family. As a native perennial herb, it is mainly grown in Korea, Japan, China, and North America [17,18,19]. Furthermore, the roots of this plant are traditionally used for medicinal purposes in the treatment of inflammation, infection, jaundice, and hyperlipidemia diseases [20].

Juglone and its derivatives from *Reynoutria japonica* have been reported to exert antimicrobial [21,22,23] and anticancer [24] activities. We previously reported that a new natural juglone isolated from this plant, 2-methoxy-7-acetonyljuglone (MA) showed potent anti-*Helicobacter pylori* activity [25]. In this study, we further investigated the effect of MA on NRF2 activation in HeLa cells. 

## 2. Materials and Methods 

### 2.1. Chemicals

Four derivatives of juglone, 2-methoxy-7-acetonyljuglone (MA), 2-ethoxy-6-acetyl-7-methyljuglone (EAM), 2-methoxy-6-acetyl-7-methyljuglone (MAM) and 2-methyl-3-acetyl-7-methoxyjuglone (MAMO) were isolated from *R. japonica* in our laboratory according to the method as previously described [25]. Anti-NRF2 (ab137550) and anti-HO-1 (ab68477) antibodies were obtained from Abcam (Cambridge, MA). A p38 MAPK inhibitor, SB203580 and a PI3K/AKT inhibitor, LY294002 were purchased from Sigma Aldrich (St. Louis, MO). U0126 of a MEK inhibitor and primary antibodies against p-p38, p38, p-AKT, AKT, p-ERK1/2, and ERK1/2 were from Cell Signaling Technology (Danvers, MA, USA). The antibodies against Lamin A/C, GFP, and GAPDH (sc-25778) used here were purchased from Santa Cruz Biotechnology (Santa Cruz, CA).

### 2.2. Cell Culture

HeLa cells (ATCC, VA, USA) were cultured with RPMI 1640 medium containing 10% fetal bovine serum and antibiotic-antimycotic (100 units/mL of penicillin, 100 µg/mL of streptomycin and 0.25 µg/mL of amphotericin B) in a humidified incubator at 37 °C, 5% CO_2_, and 95% air. Cells were grown at 60–70% confluence for sub-culturing and all experiments.

### 2.3. Cell Toxicity Assay

The MTT assay was performed to determine the cytotoxic effect of MA on HeLa cells, as previously described [26]. Briefly, cells were seeded in 48-well plates and treated with different doses of MA (0–25 μM) for 24 h. All samples were dissolved in DMSO and the final concentration did not exceed 0.2%. Then, 20 μL of MTT solution (5 mg/mL) was added to each well and incubated for 2 h. After discarding the media, 150 μL of DMSO was added into each well in order to dissolve formazan crystals in the cells. The absorbance by the purple color resulting from the formation of formazan was measured at 570 nm using a plate reader (Tecan instrument, CA, USA). All experiments were executed in triplicate and repeated at least twice.

### 2.4. ARE Luciferase Assay

The effect of MA on the ARE luciferase activity was measured in HeLa cells using Dual-Luciferase Reporter Assay (Promega) according to the manufacturer’s instructions. Briefly, cells were cultured in 48-well plates, treated with different concentrations of MA (0–5 μM) for 7 h and then lysed with 100 μL of passive lysis buffer at room temperature (22–24 °C). The lysates (10 μL) were used to measure the ARE luciferase activity. The values of the Renilla luciferase activity were used to normalize the ARE luciferase enzyme activity.

### 2.5. Western Blot Analysis

HeLa cells were cultured in 6-well plates until they reached 60–70% confluency prior to the addition of drug or DMSO (0.1%) for the indicated periods and concentrations as indicated in the figures. M-PER buffer was used for the isolation of cytosolic and nuclear proteins, and the whole-cell lysates were prepared by using RIPA buffer [27]. The bicinchoninic acid (BCA) assay was applied to measure the protein concentration using the BCA re-agent (Thermo Scientific, Waltham, MA) by reading the absorbance at 570 nm. Total proteins (25 µg) were separated on a gradient SDS-polyacrylamide gel (4–20%) and transferred onto a nitrocellulose membrane using the Trans-Blot Turbo system (Bio-Rad, Hercules, CA). After membrane blocking with 5% non-fat dry milk in PBS or TBS buffer containing 0.1% Tween-20 for 1 h, the primary antibodies (1:1000) were incubated overnight followed by incubation with the secondary antibodies (1:5000) horseradish peroxide conjugated for additional 1 h. The protein signal was visualized using an ECL substrate solution (Bio-Rad) and a chemiDoc System (Bio-Rad).

### 2.6. Real-Time PCR Analysis

After isolation of total RNA using TRIzol reagent (Invitrogen, Carlsbad, CA), cDNA (1 µg) was synthesized using SuperScript^TM^ II Reverse Transcriptase kit (Invitrogen). The PCR reaction was performed using SYBER Green PCR Master Mix (Roche, Mannheim, Germany). Thermocycler conditions were set as follows: Activation at 95 °C for 5 min; amplification for 45 cycles, including denaturation at 95 °C for 10 s, annealing at 60 °C for 10 s, and extension at 72 °C for 20 s; and cooling at 4 °C for 10 s. The following primer sets were used: *HO-1* forward, 5′-GAG TGT AAG GAC CCA TCG GA-3′ and *HO-1* reverse, 5′-GCC AGC AAC AAA GTG CAA G-3′; *NQO-1* forward, 5′-TCC TTT CTT CTT CAA AGC CG-3′, and *NQO-1* reverse, 5′-GGA CTG CAC CAG AGC CAT-3′; *GCLC* forward, 5′-CTT TCT CCC CAG ACA GGA CC-3′ and *GCLC* reverse, 5′-CAA GGA CGT TCT CAA GTG GG-3′; *NRF2* forward, 5′-TCT TGC CTC CAA AGT ATG TCA A-3′ and *NRF2* reverse, 5′-ACA CGG TCC ACA GCT CAT C-3′; *GAPDH* forward, 5′-AAG GTG AAG GTC GGA GTC AA-3′ and *GAPDH* reverse, 5′-AAT GAA GGG GTC ATT GAT GG-3′.

### 2.7. Immunocytochemistry

Immunocytochemistry for NRF2 was followed, as previously described [28]. Briefly, HeLa cells were cultured on 35-mm glass-bottom dishes and treated with MA (5 µM) for 17 h. Next, the cells were fixed in 4% paraformaldehyde for 10 min and ice-cold methanol for another 2 min. Cells were washed three times with PBST (0.3% Triton X-100) and blocked with 5% donkey serum (Abcam) in PBST for 1 h at RT followed by incubation with anti-NRF2 antibody in PBST containing 5% donkey serum and 3% BSA overnight at 4 °C. Cells were washed with PBST three times and then incubated with secondary antibody conjugated with Alexa Fluor^®^ 488 for 1 h. For nuclear staining, cells were treated with DAPI solution for 1 min. Images were captured using fluorescence microscopy (Nikon instrument).

### 2.8. Protein Stability Assay

To examine the effect of MA on the NRF2 stability, HeLa cells were treated with or without 5 μM. To observe the effect of MA on the NRF2 stability, HeLa cells were treated with or without MA (5 μM) for 4 h and incubated with cycloheximide (CHX, 5 μg/mL) for different periods. Western blot assay was performed, and the half-life of NRF2 protein was deduced from the result using densitometry and non-linear curve was made using GraphPad Prism 5 software. For ubiquitination assay, cells were cultured in 10-cm dishes and transfected with plasmids DNA of pcDNA4-His-Ubi (4 μg) and pEGFP-NRF2 (4 μg) for 24 h using polyethyleneimine. After treatment with MA (5 μM) for 4 h, the cells were collected, and then the whole-cell lysates were prepared by using a RIPA lysis buffer. Whole-cell extract (250 μg) was incubated with 50 μL of the Ni-NTA agarose slurry in 500 μL RIPA buffer for 1 h at 4 °C in a rotary shaker. After washing with RIPA buffer, the beads were resuspended in 2x Laemmli sample buffer. Samples were boiled for 5 min and then subjected to SDS-PAGE for the western blotting assays.

### 2.9. Statistical Analysis

All data were expressed in mean ± SD (standard deviation). Statistical analysis was carried out using the two-tailed Student’s *t*-test for unpaired data. Values of *p* < 0.05 were considered statistically significant.

## 3. Results

### 3.1. MA Increases the NRF2 Activity through ARE System in HeLa Cells

**Figure 1:**
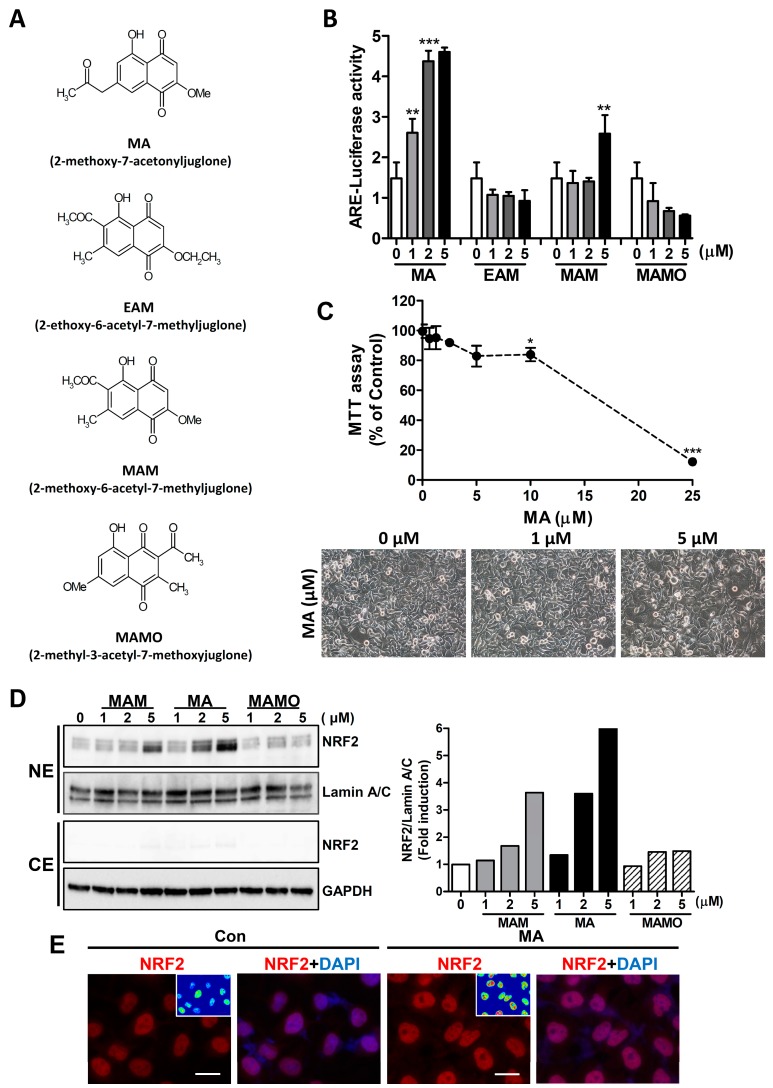
MA increases the ARE luciferase activity and nuclear accumulation of NRF2 in HeLa cells. (**A**) Chemical structures of juglone derivatives. (**B**) The ARE-luciferase activity was measured in HeLa cells, after treatment with indicated concentrations of juglone derivatives for 7 h. ** *p* < 0.001, *** *p* < 0.0001. (**C**) The representative images of the HeLa cell and MTT assay after treatment with different concentration of MA for 24 h. * *p* < 0.05, *** *p* < 0.0001. (**D**) Western blot analysis of NRF2 after treatment with juglone derivatives for 16 h. Densitometric analysis of NRF2 is shown in the right panel. NE, nuclear extract; CE, cytosolic extract. (**E**) Immunocytochemistry for NRF2 by MA (5 μM). Scale bar, 20 μm.

To examine the effect of juglone derivatives on NRF2 activity, the ARE luciferase activity was measured in HeLa cells after transfection of the reporter plasmid (Figure 1A). Among the juglone derivatives, 2-methoxy-7-acetonyljuglone (MA) strongly stimulated the luciferase activity in a dose-dependent manner (Figure 1B). Hence, MA was selected for further study to elucidate the mechanism. To determine the cytotoxicity of MA, HeLa cells were treated with different concentration of MA (0–25 μM) for 24 h. The results showed that MA exhibited a strong cytotoxic effect at 25 μM. However, the results of MTT assay and cell morphology showed that the cytotoxic effect of MA at 5 μM was minimal, whereas the ARE-luciferase activity was strongly induced at this concentration (Figure 1C). Since active NRF2 is largely localized in the nucleus, then the level of NRF2 in the nucleus was measured by western blot analysis and immunocytochemistry. MA showed a significant increase in nuclear accumulation of NRF2 in a dose-dependent manner for 16 h in both western blot (Figure 1D) and immunocytochemistry analyses (Figure 1E).

### 3.2. MA Increases NRF2 Target Genes in HeLa Cells

**Figure 2:**
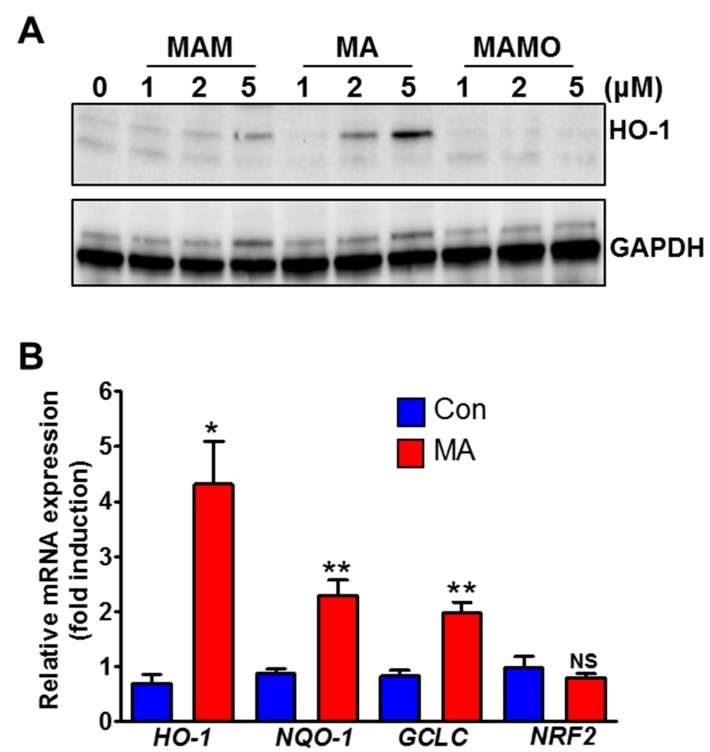
MA increases NRF2 target genes in HeLa cells. (**A**) Western blot analysis of HO-1 by juglone derivatives. (**B**) Real time-PCR analysis of NRF2 target genes after treatment with MA (5 μM) for 17 h. * *p* < 0.05, ** *p* < 0.001.

To confirm the effect of MA on the NRF2 activity, the expression of the NRF2-target genes and proteins was measured using qRT-PCR. Heme oxygenase-1 (HO-1), a well-known NRF2 target protein, was strongly increased by MA compared to other juglone derivatives (Figure 2A). In addition, the mRNA levels of *HO-1*, *NQO-1*, and *GCLC* genes were significantly induced upon the treatment of MA (Figure 2B). However, no change in the level of *NRF2* mRNA was observed by MA, which suggests that the induced expression of the NRF2 target genes is attributed to the NRF2 stability and activity.

### 3.3. MA Increases NRF2 Activity through p38 and AKT Signaling in HeLa Cells

**Figure 3:**
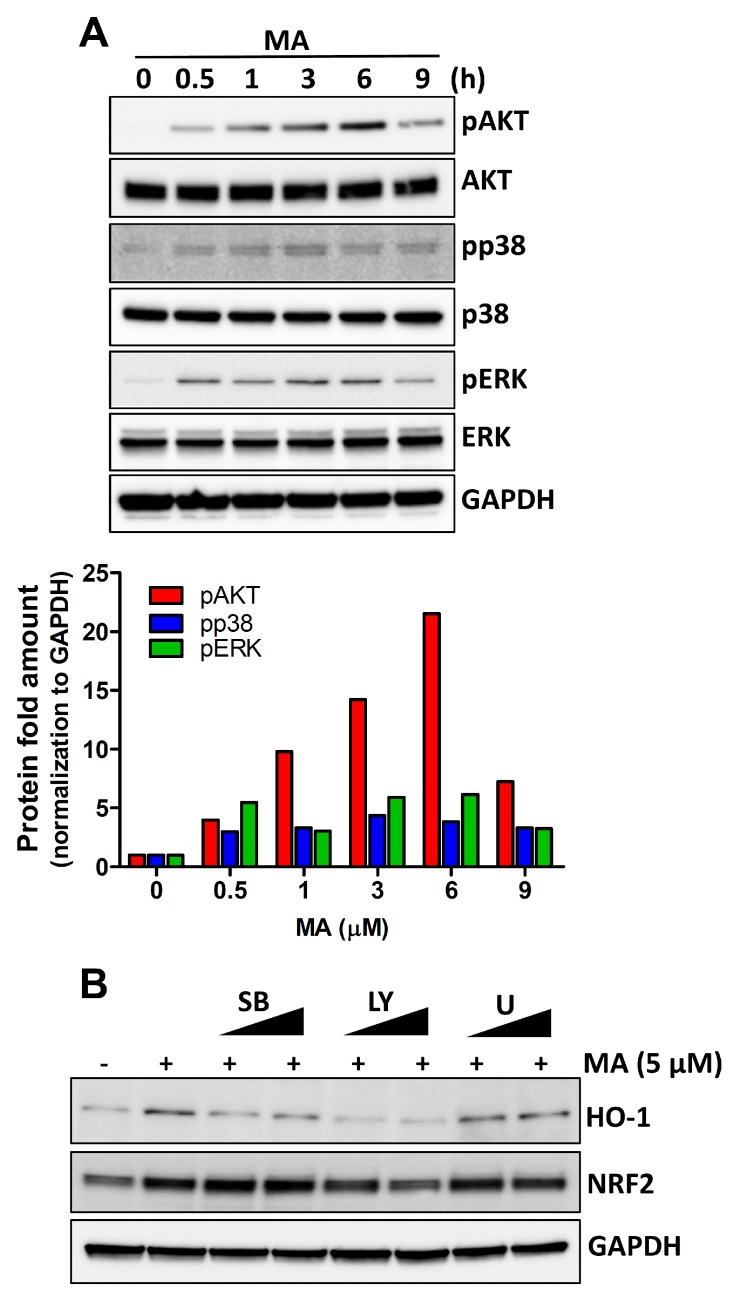
MA increases the NRF2 activity through p38 and AKT signaling in HeLa cells. (**A**) The Western blotting assay shows the activation of AKT, p38, and ERK by MA in different periods. Densitometric analysis of phosphorylated proteins is shown below (A). (**B**) Western blotting data shows the HO-1 and NRF2 levels after treatment with different inhibitors (5 μM and 10 μM) in the presence of MA for 4h. SB, a p38 inhibitor SB203580; LY, an AKT inhibitor LY294002; U, a MEK1/2 inhibitor.

To explore the underlying molecular mechanisms in the MA-induced NRF2 activation, we measured the phosphorylation of AKT, p38, and ERK using western blot analysis. Our results showed that MA increased the phosphorylation levels of p38 and AKT in a time-dependent manner. However, phosphorylation of ERK by MA persisted from 30 min to 6 h (Figure 3A). To further explore the signaling pathways involved in MA-induced NRF2 activation, we tested the effects of the pharmacological inhibitors of PI3K/AKT (LY294002), p38 (SB203580), and ERK1/2 (U0126) on the NRF2 phosphorylation. Western blot assays showed that inhibition of both p38 and AKT decreased the MA-induced HO-1 levels, albeit, inhibition of ERK did not affect the levels of HO-1. However, the level of NRF2 was affected only by AKT inhibition (Figure 3B).

### 3.4. MA Increases the NRF2 Stability through Inhibition of Ubiquitin-Mediated Degradation Pathway

**Figure 4:**
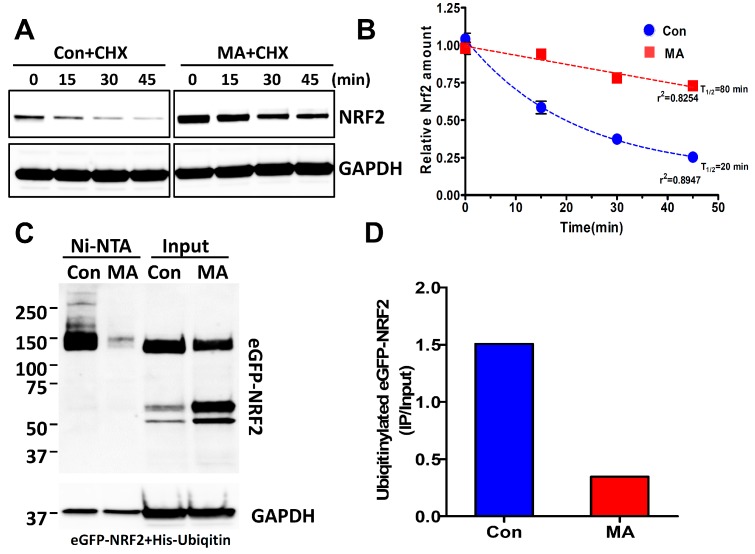
MA increases the NRF2 stability by inhibiting ubiquitin-mediated protein degradation in HeLa cells. (**A**) Cells were treated with MA (5 μM) for 4 h followed by incubation with cycloheximide (CHX) (5 μg/mL) for the indicated periods. Western blotting was accomplished with the whole protein lysates. CHX, cycloheximide. (**B**) The stability of NRF2 was analyzed using densitometer from (A). (**C**) Cells were treated with MA (5 μM) for 4 h after co-transfection of pEGFP-NRF2 and pcDNA3.1-His ubiquitin plasmids. His-ubiquitinated proteins were purified using Ni-NTA agarose beads. Ubiquitinylated eGFP-NRF2 was analyzed by a western blot assay. (**D**) The quantity of ubiquitinylated eGFP-NRF2 was measured using a densitometer from (C).

As shown in Figure 2B, MA did not affect the level of *NRF2* mRNA. Since NRF2 protein is degraded by the proteasomal degradation pathway using the Keap1-Cul3 system [29], we tested whether MA could affect the NRF2 stability. Cells were treated with cycloheximide (5 μg/mL) for 4 h after treatment with MA. NRF2 induced by MA persisted up to 45 min (Figure 4A). Figure 4B shows the analysis of the half-life of NRF2 protein. MA significantly prolonged the NRF2 half-life when compared with the control. Furthermore, to confirm whether MA inhibited NRF2 ubiquitination, the Ni-NTA purification system was employed to purify His-tagged-ubiquitinylated-NRF2 from eGFP-NRF2- and His-Ubiquitin-expressing HeLa cells after transfection of these plasmids. We observed that the levels of ubiquitinated eGFP-NRF2 were significantly decreased in the presence of MA (Figure 4C,D), suggesting that MA inhibited the ubiquitination of eGFP-NRF2 and consequently increased the NRF2 stability.

## 4. Discussion

In the present study, we provided several lines of evidence for the stimulatory effect of a juglone derivative MA on the ARE luciferase activity and NRF2 nuclear accumulation in HeLa cells. In addition, HO-1 is a downstream regulator of NRF2 activity, and its levels significantly increased in MA-treated cells. The levels of NRF2 were induced within 4 h after MA treatment and lasted up to 16 h. In addition, mRNA levels of NRF2 target genes, including *HO-1*, *NQO-1*, and *GCLC* were also increased upon MA treatment. Furthermore, MA increased the NRF2 activity via p38 and AKT signaling, as well as stabilization of NRF2. Therefore, it is suggested that MA could modulate the oxidative stress by promoting NRF2 activity in the cells.

As mechanisms for NRF2 activation are concerned, MAPK and PI3K/Akt have been implicated in translocation of NRF2 to the nucleus [4]. In this study, we analyzed the possible signaling mechanism using specific inhibitors for MAPK and PI3K/Akt. Interestingly, p38 inhibitors failed to inhibit the NRF2 levels in the presence of MA. However, it did inhibit HO-1 levels. We speculated that p38 inhibitor might block the NRF2 translocation to the nucleus. In addition, AKT inhibitor decreased the levels of both NRF2 and HO-1 proteins. We reasoned that AKT might play an important role in the degradation of NRF2. Because ubiquitinated NRF2 can be degraded by the 26S proteasome complex containing Keap1 and Cul3 [8,30], blocking of ubiquitination by specific compounds would increase the NRF2 function. Natural chemicals such as mangiferin [31] and methoxycinnamoyl-α-l-rhamnopyranosyl ester (MCR) [32] were found to inhibit NRF2 ubiquitination and increase NRF2 protein stability. In conclusion, our study indicates that MA promotes the NRF2 accumulation in the nucleus where NRF2 binds to the ARE of its target genes and stimulates the expression of these genes.

In medicinal plants, naphthoquinone, including juglone and its derivatives, exhibit antibacterial activity [21,33]. In the previous report, MA, among other juglone derivatives, showed a strong anti-*H. pylori* activity [25]. Since oxidative stress induced by *H. pylori* can lead to damage of gastric epithelial cells [34], we conclude that MA can be used for chemoprevention or chemotherapeutic purposes.
